# A simple manoeuvre to minimise bladder injury during laparoscopic incisional hernia repair

**DOI:** 10.1308/003588412X13373405385214p

**Published:** 2012-07

**Authors:** DL Sanders, JP Evans, I Finlay

**Affiliations:** Royal Cornwall Hospitals NHS Trust,UK

## BACKGROUND

Bladder injury during laparoscopic incisional hernia repair has a reported incidence of 0.5%.^1^ Mesh placement during repair may be difficult when the fascial defect extends towards the pubis. The bladder may need to be separated from the peritoneum in order to place the mesh safely and achieve adequate mesh overlap.^2^ We propose a simple intra-operative technique to help identify bladder position and better define the plane for dissection.

## TECHNIQUE

A transurethral Foley catheter is inserted. Prior to pelvic dissection, the bladder is filled with 400ml of normal saline via the catheter. As the bladder fills, it projects upward out of the pelvis into the abdomen and defines the plane for dissection. The bladder should now be within the surgical field and easily identifiable.

## DISCUSSION

The above technique provides a simple way to confirm bladder position laparoscopically. Once visualised within the surgical field, further dissection, mesh placement and safe mesh fixation avoiding the bladder can proceed. Furthermore, where iatrogenic bladder injury is suspected, the technique also provides direct visualisation of any leak through retrograde methylene blue dye instillation.^3^
